# Magnetic resonance-guided focused ultrasound surgery for adenomyosis: current evidence and future directions

**DOI:** 10.3389/fmed.2026.1794748

**Published:** 2026-05-14

**Authors:** Yang Li, Zhengguang Yang, Xunhua Xu

**Affiliations:** 1 Department of Radiology, China Resources and WISCO General Hospital, Wuhan University of Science and Technology, Wuhan, China; 2 School of Medicine, Wuhan University of Science and Technology, Wuhan, China

**Keywords:** ablation, adenomyosis, fertility preservation, high-intensity focused ultrasound, magnetic resonance imaging, magnetic resonance-guided focused ultrasound surgery

## Abstract

Adenomyosis (AM) is a common gynecological disorder with high prevalence among women of childbearing age. Although hysterectomy is the definitive treatment, it is considered unsuitable for patients who wish to preserve fertility, creating an urgent need for effective, minimally invasive alternatives. Despite emerging evidence, no comprehensive synthesis comparing ablation techniques and guidance modalities specifically for AM currently exists. Currently used thermal ablation techniques include high-intensity focused ultrasound (HIFU), radiofrequency ablation, and percutaneous microwave ablation. Among these, HIFU has gained increasing attention as a non-invasive, uterus-sparing treatment option. Focused ultrasound procedures can be performed under ultrasound or magnetic resonance imaging (MRI) guidance. Magnetic resonance-guided focused ultrasound surgery (MRgFUS), leveraging the high resolution of MRI, enables more precise lesion targeting, real-time temperature monitoring, and immediate postoperative assessment. MRgFUS can effectively reduce lesion size and alleviate symptoms, while lesion-specific MRI features and radiomics models show predictive value for treatment outcomes. By integrating adjuvant hormonal therapies and advanced imaging evaluations, MRgFUS is expected to not only evolve into a more individualized and precise therapeutic strategy but also help establish standardized frameworks for patient selection and outcome prediction. This review summarizes recent advances in MRgFUS for the treatment of AM, focusing on its unique advantages, safety, and efficacy, and emphasizes the critical role of MRI-based imaging biomarkers in predicting therapeutic outcomes.

## 1 Introduction

Adenomyosis (AM), a benign uterine disorder of adult women, is characterized by the presence of endometrial glands and stroma deep within the myometrium (1). Its clinical manifestations include menorrhagia, dysmenorrhea, irregular uterine bleeding, and infertility (2). Historically, the condition has often been underdiagnosed and usually identified only after a hysterectomy (3). However, advances in imaging have improved detection, with prevalence estimates of 21–34% among women of reproductive age (4). Moreover, AM frequently coexists with other gynecological conditions, such as endometriosis and uterine fibroids (UF), which complicate both diagnosis and management (5). In addition, AM is associated with adverse reproductive outcomes, including reduced pregnancy rates and increased miscarriage risk (6). Thus, effective treatments with minimal adverse effects are warranted.

Although hysterectomy remains the definitive treatment, it is considered unsuitable for women who wish to preserve fertility and carries inherent surgical risks (7). Thermal ablation is a minimally invasive alternative, encompassing radiofrequency ablation (RFA), high-intensity focused ultrasound (HIFU) ablation, and percutaneous microwave ablation (PMWA), each with different characteristics (8). Introduced in the early 1990s, HIFU is a non-invasive treatment technique that focuses ultrasonic energy on target tissues, raising temperatures above 60 °C to induce coagulation necrosis (9, 10). Currently, HIFU treatment can be divided into ultrasound-guided focused ultrasound surgery (USgFUS) and magnetic resonance-guided focused ultrasound surgery (MRgFUS) (11).

**Figure 1:**
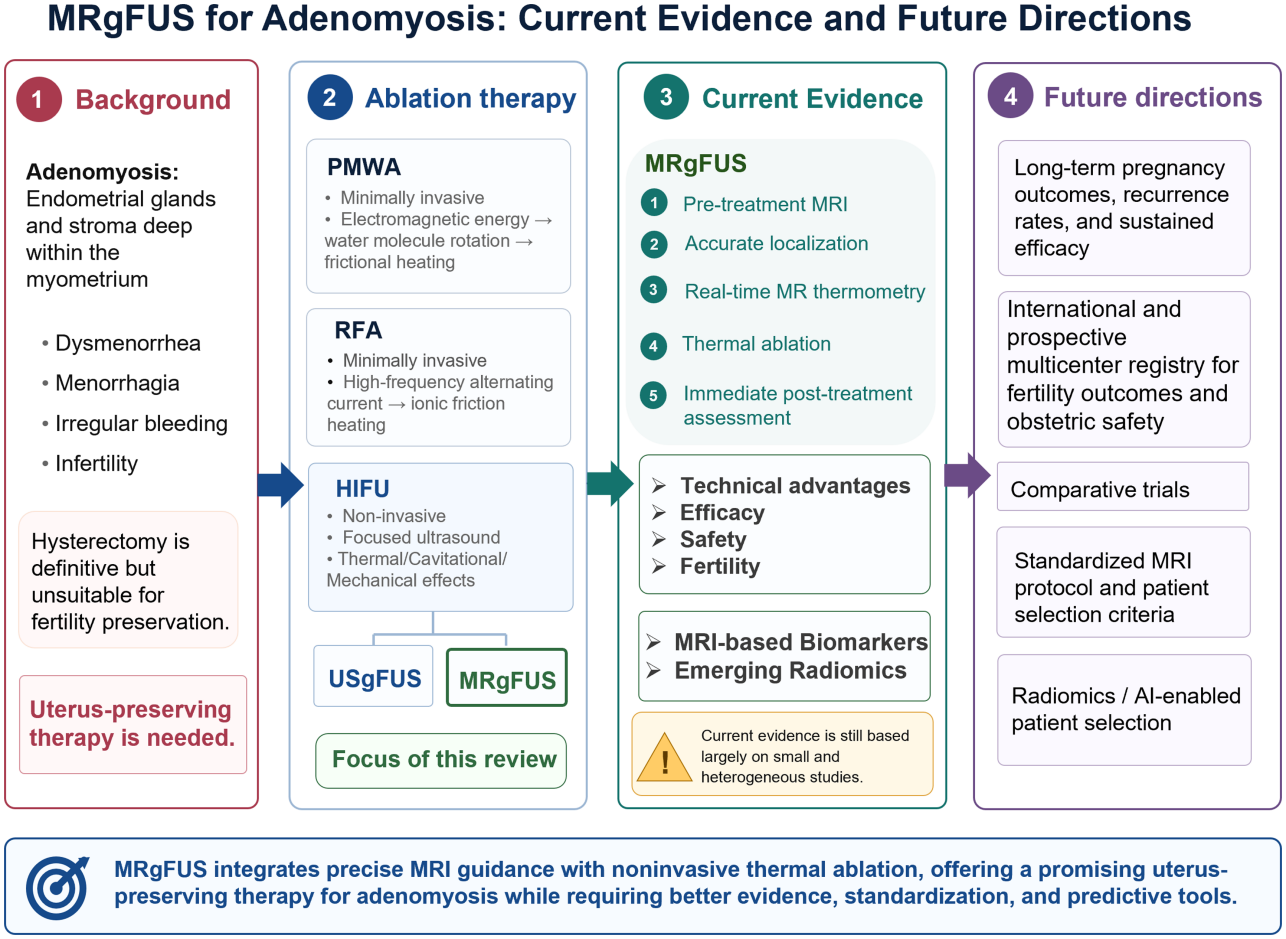
Conceptual framework of this review. The diagram outlines the clinical background, ablation landscape, current evidence, and future directions of MRgFUS for AM.

In recent years, MRgFUS has achieved significant results in treating UF (12–14). Its application in the field of AM is gradually receiving attention (15, 16). However, despite emerging evidence, there is currently no comprehensive synthesis comparing ablation techniques and guidance modalities specifically for AM. This review compares three ablation techniques and two guidance modalities, systematically summarizing the advantages of MRgFUS and recent advances in its use for AM, to provide clinical insights and identify future research directions. The overall framework is summarized in Figure 1.

## 2 Ablation therapy for AM

HIFU, PMWA, and RFA are the primary ablation methods for treating AM. PMWA relies on ultrasound-guided percutaneous insertion of electrodes directly into the lesion, where electromagnetic energy rapidly rotates adjacent polar water molecules for heat generation (17). Similarly, under ultrasound guidance, an RFA needle is inserted directly into the lesion, and a high-frequency alternating current generates heat through ionic friction, thereby inducing tissue necrosis (18).

Both PMWA and RFA are invasive procedures and carry inherent risks, including thermal spread and mechanical trauma, which may affect endometrial integrity and fertility function (19). The advantages of PMWA include its shorter operative time, larger ablation volume, high ablation efficiency, and ability to maintain consistently higher tissue temperatures (20). However, these benefits also entail a higher risk of thermal injury, with its sensitivity varying based on the tissue type (21, 22). Currently, certain novel methods used to reduce treatment-related injuries, such as artificial ascites and the instillation of intrauterine chilled saline, have demonstrated promising results (23, 24). RFA exhibits favorable ablation time and lesion volume reduction; however, the inability to monitor temperature in real time increases the risk of thermal damage to the endometrium and intrauterine adhesions development (25–27). Despite these risks, preliminary evidence has suggested that RFA offers advantages in terms of lower recurrence rates (28). Overall, PMWA and RFA exhibit similar safety and efficacy profiles for treating symptomatic AM (29). Moreover, these ablation techniques have the potential to preserve fertility, and RFA has shown promising fertility outcomes (30, 31). Nevertheless, rare but serious complications, such as uterine rupture during pregnancy following RFA (32), have been reported, indicating that the safety of these techniques, particularly regarding fertility outcomes, warrants further investigation.

In contrast, HIFU is a non-invasive technique that offers fertility preservation (33, 34). HIFU primarily targets the superficial layer of the lesion; therefore, treatment outcomes are influenced by lesion location, blood supply, and tissue characteristics (35). Despite requiring a longer treatment time, a single HIFU session achieves significant symptom relief while preserving uterine structural and functional integrity and ovarian function (8, 36). Therefore, HIFU is a viable treatment option, especially for patients prioritizing fertility preservation.

## 3 Comparison of MRgFUS and USgFUS

Although both MRgFUS and USgFUS use HIFU for generating thermal effects that induce coagulative necrosis in target tissues, the core difference in their imaging guidance methods underlies the distinct clinical outcomes of the two techniques.

### 3.1 Technical aspects

In terms of imaging and targeting, high spatial resolution MRI allows MRgFUS to clearly visualize deep anatomical structures and provide accurate three-dimensional localization, minimizing the risk of off-target tissue damage (37). Moreover, MR image quality shows no deterioration as HIFU sonications are repeated, which is one of the drawbacks of USgFUS. Although MRgFUS is more susceptible to motion and has lower temporal resolution, this is not clinically problematic: current systems achieve a refresh rate of 2–3 s for magnitude images and thermometric measurements (38), which is sufficient for real-time guidance.

In contrast, USgFUS relies on two-dimensional ultrasound imaging, which is prone to positional deviations in deep lesions. Accurately delineating the exact lesion boundaries and extent remains challenging for diffuse AM with ill-defined borders (39). This may lead to incomplete ablation or injury to adjacent organs. Correlational studies have revealed that the non-perfused volume ratio (NPVR) is significantly lower in diffuse AM than in focal AM, partly due to restricted ablation ranges implemented for safety (40). Additionally, image quality during USgFUS may be compromised by patient body habitus or bowel gas, and procedural outcomes are highly operator dependent (41). MRgFUS may be more suitable than USgFUS for treating diffuse AM, especially in extensive or ill-defined lesions (42, 43). Future studies should define safer and more extensive ablation margins for enabling complete treatment of diffuse lesions.

A critical distinction lies in real-time monitoring. MRgFUS uses magnetic resonance thermometry to generate real-time temperature distribution maps for thermal dose estimation (44). This allows dynamic adjustment of ultrasound energy and intraoperative prediction of thermal damage, ensuring complete coagulative necrosis of the target area (45). Furthermore, MR thermometry is beneficial for research because it makes quantitative analysis feasible. In contrast, USgFUS relies on real-time anatomic imaging, displaying hyperechogenic changes in tissues during and after ablation (46). Nonetheless, tissue damage can only be indirectly assessed through grayscale variations. These grayscale changes reflect post-coagulation echogenic alterations rather than real-time thermal dose deposition, contributing to a lag in adjusting ultrasound energy (47).

In terms of treatment efficiency and cost, USgFUS offers clear advantages, with a treatment duration of approximately 1 h and lower equipment costs, making it more accessible for widespread clinical use (48). In contrast, MRgFUS requires 2–3 h per session and incurs expensive equipment and higher operational costs (49, 50). However, to date, no studies have specifically investigated the cost-effectiveness of MRgFUS for AM, and reimbursement for this indication is not yet widely available, underscoring the need for further health economic evaluations to support its broader clinical implementation.

Regarding treatment assessment, MRgFUS offers multiparameter evaluation throughout the procedure: preoperative identification of active lesion areas through T2-weighted imaging (T2WI) and diffusion-weighted imaging (DWI); intraoperative dynamic contrast-enhanced (DCE) sequences to monitor microvascular permeability changes; and immediate postoperative evaluation of ablation rate using quantitative NPVR (51, 52). These objective imaging biomarkers enhance efficacy prediction. USgFUS assessment primarily relies on intraoperative grayscale observation and postoperative MRI verification, which is more subjective and delays efficacy verification (53).

### 3.2 Efficacy

In terms of efficacy, direct comparative studies comparing MRgFUS with USgFUS are currently lacking, and the reported outcomes are influenced by the study design and adjuvant therapy strategies. MRgFUS has demonstrated favorable effects in reducing lesion volume and relieving dysmenorrhea and menorrhagia (37, 42, 43), with sustained symptom relief observed at 6 and 18 months (54, 55). In an early small-sample study, Fan et al. (56) have reported a mean immediate post-treatment NPVR of 62.5% for MRgFUS. With gonadotropin-releasing hormone agonist (GnRH-a) pretreatment, Huang et al. (57) have achieved a mean NPVR of 83.3%, along with a 47.9% reduction in the pictorial blood loss assessment chart score and a 15.3% increase in hemoglobin at 12 months. Data are more abundant for USgFUS. Wu et al. (16) have reported a mean NPVR of 69.77% for USgFUS lesion ablation alone, with a 36.2% effectiveness rate for menorrhagia at 12 months post-treatment. Li et al. (49) have conducted a large-sample study and have reported a mean NPVR of 64.5% for USgFUS alone. The effectiveness rate for dysmenorrhea decreased from 89.0% at 3 months to 65.8% at 5 years, and combining with the levonorgestrel-releasing intrauterine system (LNG-IUS) significantly improved long-term outcomes. These data indicate that both guidance modalities can achieve satisfactory therapeutic effects; however, combination with adjuvant therapy is a key strategy for improving ablation efficacy and long-term outcomes.

### 3.3 Safety

Regarding safety, the two guidance modalities have demonstrated similar rates of common adverse events (58). However, MRgFUS offers a theoretical advantage in avoiding severe complications, such as bowel or nerve injury, due to its real-time thermometry, which enables precise control of ablation margins (56). In contrast, USgFUS relies on intraoperative patient feedback and grayscale changes to maintain a low incidence of severe complications (59). Future comparative studies are warranted to clarify the safety profiles of these two guidance modalities.

A large-scale safety analysis of HIFU by Lu et al. (58) provides valuable insights. In their evaluation of patients with UF or AM, postoperative adverse events were common but mostly mild and self-limiting. Major complications were rare (0.15%), including uterine rupture, necrotic tissue obstruction requiring surgery, third-degree skin burns, and persistent lower limb pain or movement disorders. The study has emphasized that the majority of adverse events can be effectively prevented and controlled through rigorous patient selection, meticulous intraoperative techniques (such as controlling ablation margins and real-time temperature monitoring), and standardized postoperative management.

Although rare, uterine rupture warrants high vigilance. Wu et al. (60) have reported a case of spontaneous conception 7 years after HIFU, in which the patient experienced sudden full-thickness uterine rupture at 34 weeks of gestation; the rupture site corresponded exactly to the ablation zone from 7 years earlier, and retrospective review indicated that the ablation had breached the uterine serosa. Liu et al. (61) have reported a case of a patient who underwent aggressive HIFU ablation (with the ablation margin <1.0 cm from both the endometrium and serosa) due to the absence of fertility desire. However, an unplanned pregnancy occurred 8 months after treatment, and at 38 weeks of gestation, the uterus was found to have only an intact serosal layer remaining.

Based on current evidence, risk factors for uterine rupture after HIFU include full-thickness ablation, serosal breach, excessive ablation volume, short treatment-to-pregnancy interval (as short as 4 months), and prior cesarean section (11, 36). Therefore, full-thickness ablation should be strictly avoided for patients desiring future fertility, with a recommended residual myometrial thickness of at least 1 cm. An interval of at least 6–12 months between treatment and pregnancy is recommended, along with close monitoring during pregnancy.

### 3.4 Fertility

In terms of fertility outcomes, research data on MRgFUS are limited, with existing reports mentioning only seven pregnancies and five live births (11). Although MRgFUS may not impair ovarian reserve (15), this finding was based on only five patients with AM; therefore, larger studies are warranted for confirmation. In contrast, research on the fertility outcomes of AM using USgFUS is relatively more abundant. A study on pregnancy outcomes has reported an overall postoperative pregnancy rate of 38.8%, with the highest rate observed in patients with endogenous AM (36, 62). Meanwhile, a multiple regression analysis revealed that age <35 years, anti-Müllerian hormone levels >3.35 ng/mL, the presence of lesions in the posterior wall, and endogenous AM are independent predictors of successful pregnancy (62). However, rare cases of uterine rupture during pregnancy have been reported in patients who have undergone USgFUS (60, 61). Overall, evidence on fertility outcomes of the two guidance methods remains insufficient.

Table 1 summarizes the key differences among the ablation techniques. A critical conclusion from this comparison is that all four techniques are effective and safe as a whole. Overall, while these techniques offer benefits, more high-quality randomized controlled trials are warranted to directly compare them and clarify their respective advantages.

**Table 1:** Comparative analysis of ablation modalities for AM.

Category	MRgFUS	USgFUS	RFA	PMWA
Principle	Focused ultrasound energy produces thermal, cavitational, and mechanical effects	Same as left	High-frequency alternating electrical current generates heat by ionic friction	Electromagnetic energy rotates polar water molecules to generate heat
Invasiveness	Non-invasive	Non-invasive	Minimally invasive	Minimally invasive
Guidance	MRI	Ultrasound	Ultrasound	Ultrasound
Advantages	High spatial resolution; more accurate targeting; real-time temperature monitoring; immediate efficacy assessment	Real-time anatomic imaging; low cost; short treatment time (approximately 1 h)	Shorter ablation time (average 37.5 min) (29); symptom recurrence low	Shortest ablation time (mean: 16.3 min) (29); consistent high tissue temperature; larger ablation volume
Limitations	Expensive equipment; lower temporal resolution; long treatment duration (2–3 h)	Low resolution; limited by patient bowel gas	Requires needle insertion; lack of accurate temperature monitoring post-probe implantation	Requires puncture; higher risk of thermal injury; efficacy affected by tissue water content
Efficacy (NPVR and symptomatic relief)	NPVR: 62.5% (56); 83.3% (combination GnRH-a) (57); significant relief of dysmenorrhea and menorrhagia persists for at least 6–12 months	NPVR: 69.7% (16); dysmenorrhea and menorrhagia improved significantly, but long-term efficacy declined	NPVR: 79.2%; significantly alleviate dysmenorrhea and menorrhagia	NPVR: 79.7%; similar efficacy to RFA but faster
Safety	Complication rate: 10.9%; most common: buttock pain (4.5%); skin burns: 1.8%; no major complications (11)	Major complications: 13.3% bowel injury requiring resection, nerve injury; minor: 13.3% skin burn, urinary tract infection (59); rare uterine rupture	Abdominal pain, vaginal discharge, low-grade fever (self-limiting); potential endometrial thermal injury; intrauterine adhesions; rare uterine rupture	Similar safety to RFA; rare post-treatment amenorrhea
Level of fertility outcomes research	A total of seven pregnancies, five live births, and two pregnancies were reported (11); MRgFUS may not affect ovarian reserve	26-month follow-up: pregnancy rate 38.7%, with 26 live births and 18 spontaneous abortions (62)	59 patients → clinical pregnancy rate 50%, spontaneous conception 42.7%, 24 deliveries, 12 miscarriages (30)	Ovarian function preserved (FSH, estradiol unchanged); the lack of investigation of AMH; fertility data limited

AM, adenomyosis, PMWA, percutaneous microwave ablation, RFA, radiofrequency ablation, USgFUS, ultrasound-guided focused ultrasound surgery, MRgFUS, magnetic resonance–guided focused ultrasound surgery, MRI, magnetic resonance imaging, NPVR, non-perfused volume ratio, FSH, follicle-stimulating hormone, AMH, anti-Müllerian hormone.

## 4 Influencing factors and predictive methods of MRgFUS efficacy in AM

Although MRgFUS is effective for AM, its therapeutic efficacy is affected by multiple patient- and lesion-related factors. MRgFUS is primarily indicated for symptomatic patients with AM who wish to preserve their fertility (11, 63). Severe obesity and thick abdominal adipose tissue may hinder ultrasound penetration and reduce treatment effectiveness (11, 64). Moreover, a history of uterine surgery and a scar width > 10 mm can easily lead to skin burns, which may complicate treatment or recovery (65, 66).

Lesion location and morphology can affect treatment outcomes and prognosis. Zhao et al. (67) have compared the clinical efficacy of HIFU across MRI-classified AM subtypes and found that the technique is safe and effective for all types, with type III (nodular) AM showing the best therapeutic effect. These findings further confirm the effectiveness of MRgFUS in both diffuse and localized AM. Using a different MRI classification system (internal, external, full-thickness, and intramural), Gong et al. (68) have reported that internal AMs, lesions confined to the thickened junctional zone with preservation of the outer myometrium, were associated with better pain relief, whereas asymmetric external AM required higher energy for ablation. Keserci et al. (69) have proposed a novel AM classification method based on T1 perfusion-derived time–signal intensity (SI) curves of AM lesions relative to the surrounding myometrium. This approach demonstrated clinical utility in predicting immediate MRgFUS outcomes. Patients whose AM lesions exhibited time–SI curves lower than those of the myometrium appeared to benefit from MRI-guided ablation. Overall, nodular AM (type III), internally located lesions, and low T1 perfusion signals are associated with more favorable therapeutic responses.

T2WI signal characteristics are key imaging predictors of ablation efficacy. Early studies by Fukunishi et al. (54) have revealed that AM lesions with low signal intensity on T2WI were more likely to undergo effective ablation. This could be attributed to lower water content within the lesion, resulting in minimal heat loss (owing to the high thermal conductivity of water). Patients with lesions characterized by poor blood supply, predominantly low T2WI signal, thin abdominal walls without scars, anterior uterine wall lesions, and localized or large lesions are more likely to achieve a high NPVR (56, 65, 66). A higher NPVR reflects greater ablation efficiency and is associated with improved clinical outcomes.

However, subsequent studies have revealed a deeper association. A retrospective study has reported that therapeutic efficacy is correlated with the number of T2WI hyperintense foci within the lesion rather than overall signal intensity (70). Hyperintense foci on T2WI typically indicate ectopic endometrial tissue or cystically dilated endometrial glands with hemorrhage. A prospective study by Du et al. (71) has revealed that patients with ≤ 5 T2WI hyperintense foci required shorter treatment times and lower energy doses and exhibited lower energy efficiency factors while achieving higher ablation rates compared with patients with > 5 foci. These findings indicate that the number of T2WI hyperintense foci is a factor affecting the ablation rate and clinical efficacy of HIFU treatment in women with AM and may be a predictor of the efficacy of HIFU treatment for AM.

The mechanistic basis for these observations involves two complementary biophysical effects. First, the presence of numerous (> 5) hyperintense foci—which histopathologically correspond to islands of ectopic endometrial tissue, cystically dilated glands, or focal hemorrhages—creates structural inhomogeneities within the adenomyotic lesion. These tissue interfaces increase the absorption, reflection, and scattering of the incident ultrasound beam, thereby reducing the net energy deposited at the target focal zone and lowering ablative efficiency (70). Second, these hyperintense foci are typically surrounded by a rich capillary network, which enhances the heat-sink effect: blood flow rapidly convects thermal energy away from the ablation zone, further attenuating the temperature elevation required for coagulative necrosis (71). Therefore, comprehensive evaluation integrating both overall T2WI signal intensity and the number of hyperintense foci is necessary to accurately predict therapeutic outcomes and optimize patient selection for MRgFUS.

Early studies have relied on gadolinium-enhanced MRI to evaluate NPVR (54). More recently, DWI has been proposed as an alternative for preliminary assessments (72). Comparative analyses have demonstrated good agreement between DWI and DCE in measuring necrotic lesion areas. However, DWI may slightly overestimate NPVR due to T2-related effects such as postoperative edema and congestion. Nevertheless, DWI is useful for intraoperative real-time monitoring. Sainio et al. (73) have further demonstrated that a lower pretreatment apparent diffusion coefficient (ADC) was significantly associated with higher post-treatment NPVR. Thus, the integration of DWI and ADC mapping into the preoperative MRgFUS evaluation protocol is recommended.

Recent advances in radiomics offer new insights for improving outcome prediction using thermal ablation for AM. Li et al. (74) have first developed a radiomics model based on T2WI. When combined with clinical parameters, the model achieved an area under the curve (AUC) of 0.81 in the test set, significantly outperforming the clinical model alone (AUC 0.49). This finding suggests that T2WI-based radiomics features can effectively identify patients with poor symptom relief following MRgFUS. However, the study was limited by its relatively small sample size and the use of a single imaging sequence.

Radiomics research on HIFU for AM is more extensive than that on MRgFUS. Liu et al. have conducted a series of studies that progressed from a single-sequence T2WI model (AUC 0.83) to a dual-sequence T2WI + contrast-enhanced T1WI model (AUC 0.86) and innovatively predicted the energy efficiency factor to guide treatment dose planning (75–78). However, these studies are limited by small sample sizes, lack of external validation, and inconsistent efficacy thresholds. Sun et al. (79) have used a dual-sequence T2FS + contrast-enhanced T1WI model and found that when predicting NPVR ≥ 50%, the AUC reached 0.84, with clinical net benefit superior to that of single-sequence models. These studies confirmed the added value of dual-sequence information. Collectively, the influencing factors described in Table 2 form a comprehensive evaluation framework. These findings emphasize that MRgFUS is not suitable for all patients with AM and that its efficacy largely depends on strict patient selection.

**Table 2:** Factors affecting the efficacy of MRgFUS in treating AM.

Category	Details	Level of evidence^*^	Related literature
Patient factors	Symptomatic AM	4	(11)
	Fertility desire	5	(11)
	Thin abdominal walls (<4 cm subcutaneous fat)	2–3	(11, 57)
	History of uterine surgery and abdominal scar (scar width <10 mm)	3	(65, 66)
Lesion morphology	Lesion location (anterior uterine wall)	3	(66)
	MRI subtype: type III (nodular)	3	(67)
	MRI subtype: internal type (confined to thickened junctional zone, outer myometrium intact)	3	(68)
	Lesion size >2 and <10 cm	5	(11)
	Lesion thickness >3 cm	5	(66)
MRI-based biomarkers	Poor lesion vascularity	3	(66)
	T1 perfusion pattern: time-SI curve lower than myometrium	2	(69)
	T2WI signal intensity: overall predominantly low signal	2	(54)
	T2WI: internal hyperintense foci ≤5	2	(70, 71)
	DWI: large area of central low signal with a complete high-signal ring, lower ADC	2–3	(72, 73)
Emerging directions	Radiomics-based predictive models	3	(74–79)

* Evidence levels were assigned based on the Oxford 2011 criteria, as detailed in Knorren et al.’s study (11) (2024, Appendix).

AM, adenomyosis, MRgFUS, magnetic resonance–guided focused ultrasound surgery, MRI, magnetic resonance imaging, NPVR, non-perfused volume ratio, DWI, diffusion-weighted imaging, SI, signal intensity, ADC, apparent diffusion coefficient, T2WI, T2-weighted imaging.

## 5 Discussion

HIFU is less invasive than PMWA and RFA. Compared with USgFUS, MRgFUS may offer greater accuracy and safety. The core advantage of MRgFUS is the combination of HIFU ablation and MRI guidance. This synergy enables precise lesion targeting, real-time thermal monitoring, and immediate postoperative assessment. This combined approach enhances treatment efficacy and safety, including sustained symptom relief, lesion volume reduction, and maximized fertility preservation. However, despite its promise as a uterus-preserving option for patients with AM desiring future fertility, current evidence on reproductive outcomes remains limited.

Given that treatment efficacy is influenced by a constellation of patient- and lesion-specific factors, accurate patient selection remains paramount for optimizing MRgFUS outcomes. In addition to conventional predictors such as lesion location, T2WI signal characteristics, and vascularity, emerging evidence highlights the role of advanced imaging biomarkers. Most notably, radiomics models enhance patient selection and outcome prediction by transforming qualitative imaging features into quantitative biomarkers, capturing lesion heterogeneity, predicting ablation efficacy, and guiding individualized ablation dosing. Further research is warranted to investigate the multifactorial mechanisms influencing treatment outcomes, establish more standardized patient selection criteria, and promote the broader clinical adoption of this technology.

Despite the promising role of MRgFUS, several critical research gaps remain. First, existing studies are predominantly small-sample, retrospective analyses, leading to a lack of robust data on the long-term pregnancy outcomes, recurrence rates, and sustained efficacy. To address this, an international, prospective, multicenter registry is urgently required to systematically document fertility outcomes and obstetric safety following MRgFUS for AM. Second, a paucity of high-quality comparative research exists. Well-designed randomized controlled trials comparing MRgFUS with first-line medical therapies, such as GnRH-a or LNG-IUS, are essential for determining its relative efficacy, cost-effectiveness, and impact on quality of life. Meanwhile, although hormonal therapy may serve as an adjunct to MRgFUS to enhance and sustain therapeutic effects (80–82), its specific role in optimizing MRgFUS treatment outcomes remains unclear. In parallel, trials comparing MRgFUS with USgFUS and other minimally invasive surgical options are needed to clarify the clinical advantages conferred by MRI guidance. Finally, to enable significant comparative research and the development of robust predictive models, the field must establish a standardized MRI protocol, including specific sequences and timing for pre- and post-MRgFUS evaluation. Such standardization would accelerate effective radiomics research and ultimately lead to more personalized and precise patient selection criteria.
