# Supplementary material for: Magnetic resonance-guided focused ultrasound surgery for adenomyosis: current evidence and future directions
Source: Front Med (Lausanne). 2026 May 14;13:1794748. doi: 10.3389/fmed.2026.1794748 (PMC13216498; doi:10.3389/fmed.2026.1794748)

Supplementary Material

# Supplementary Figures and Tables

**Supplementary Table 1.**

Table 1. Comparative analysis of ablation modalities for AM

| **Category** | **MRgFUS** | **USgFUS** | **RFA** | **PMWA** |
| --- | --- | --- | --- | --- |
| Principle | Focused ultrasound energy produces thermal, cavitational, and mechanical effects | Same as left | High-frequency alternating electrical current generates heat by ionic friction | Electromagnetic energy rotates polar water molecules to generate heat |
| Invasiveness | Non-invasive | Non-invasive | Minimally invasive | Minimally invasive |
| Guidance | MRI | Ultrasound | Ultrasound | Ultrasound |
| Advantages | High spatial resolution; More accurate targeting; Real-time temperature monitoring; Immediate efficacy assessment | Real-time anatomic imaging; Low cost; Short treatment time (about 1 h) | Shorter ablation time (average 37.5 min) (29); Symptom recurrence low | Shortest ablation time (mean 16.3 min) (29); Consistent high tissue temperature; Larger ablation volume |
| Limitations | Expensive equipment; Lower temporal resolution; Long treatment duration (2–3 h) | Low resolution; limited by patient bowel gas | Requires needle insertion; Lack of accurate temperature monitoring post-probe implantation | Requires puncture; Higher risk of thermal injury; Efficacy affected by tissue water content |
| Efficacy (NPVR and symptomatic relief) | NPVR:62.5%(56); 83.3%(combination GnRH-a)(57); Significant relief of dysmenorrhea and menorrhagia persists for at least 6–12 months | NPVR:69.7%(16); Dysmenorrhea and menorrhagia improved significantly, but long-term efficacy declined | NPVR: 79.2%; Significantly alleviate dysmenorrhea and menorrhagia | NPVR: 79.7%; Similar efficacy to RFA but faster |
| Safety | Complication rate: 10.9%; Most common: buttock pain (4.5%); Skin burns: 1.8%; No major complications (11) | Major complications: 13.3% –bowel injury requiring resection, nerve injury; Minor: 13.3% – skin burn, urinary tract infection (59); Rare uterine rupture | Abdominal pain, vaginal discharge, low-grade fever (self-limiting); Potential endometrial thermal injury; Intrauterine adhesions; Rare uterine rupture | Similar safety to RFA; Rare post-treatment amenorrhea |
| Level of fertility outcomes research | A total of 7 pregnancies, 5 live births, and 2 pregnancies were reported (11); MRgFUS may not affect ovarian reserve | 26-month follow-up: pregnancy rate 38.7%, with 26 live births and 18 spontaneous abortions (62) | 59 patients →clinical pregnancy rate 50%, spontaneous conception 42.7%,24 deliveries, 12 miscarriages (30) | Ovarian function preserved (FSH, estradiol unchanged); The lack of investigation of AMH; Fertility data limited |

AM: adenomyosis, PMWA: percutaneous microwave ablation, RFA: radiofrequency ablation, USgFUS: ultrasound-guided focused ultrasound surgery, MRgFUS: magnetic resonance–guided focused ultrasound surgery, MRI: magnetic resonance imaging, NPVR: non-perfused volume ratio, FSH: follicle stimulating hormone, AMH: anti-Müllerian hormone

**Supplementary Table 2.**

Table 2. Factors affecting the efficacy of MRgFUS in treating AM

| **Category** | **Details** | **Level of Evidence*** | **Related literature** |
| --- | --- | --- | --- |
| Patient Factors | Symptomatic AM | 4 | (11) |
|  | Fertility desire | 5 | (11) |
|  | Thin abdominal walls (<4 cm subcutaneous fat) | 2-3 | (11,51) |
|  | History of uterine surgery and abdominal scar (scar width <10 mm) | 3 | (66,67) |
| Lesion Morphology | Lesion location (anterior uterine wall) | 3 | (67) |
|  | MRI subtype: type III (nodular) | 3 | (68) |
|  | MRI subtype: internal type (confined to thickened junctional zone, outer myometrium intact) | 3 | (69) |
|  | Lesion size > 2 and < 10 cm | 5 | (11) |
|  | Lesion thickness > 3 cm | 5 | (67) |
| MRI-based biomarkers | Poor lesion vascularity | 3 | (67) |
|  | T1 perfusion pattern: Time-SI curve lower than myometrium | 2 | (70) |
|  | T2WI signal intensity: overall-predominantly low signal | 2 | (61) |
|  | T2WI: internal hyperintense foci ≤5 | 2 | (71,72) |
|  | DWI: large area of central low-signal with a complete high-signal ring, lower ADC | 2-3 | (73,74) |
| Emerging Directions | Radiomics-based predictive models | 3 | (75–80) |

**Evidence levels were assigned based on the Oxford 2011 criteria, as detailed in Knorren et al.* (11) *(2024, Appendix)*

AM: adenomyosis, MRgFUS: magnetic resonance–guided focused ultrasound surgery, MRI: magnetic resonance imaging, NPVR: non-perfused volume ratio, DWI: diffusion-weighted imaging, SI: signal intensity, ADC: apparent diffusion coefficient, T2WI: T2-weighted imaging

**Supplementary Figure 1.**

Figure 1. Conceptual framework of this review. The diagram outlines the clinical background, ablation landscape, current evidence, and future directions of MRgFUS for AM.


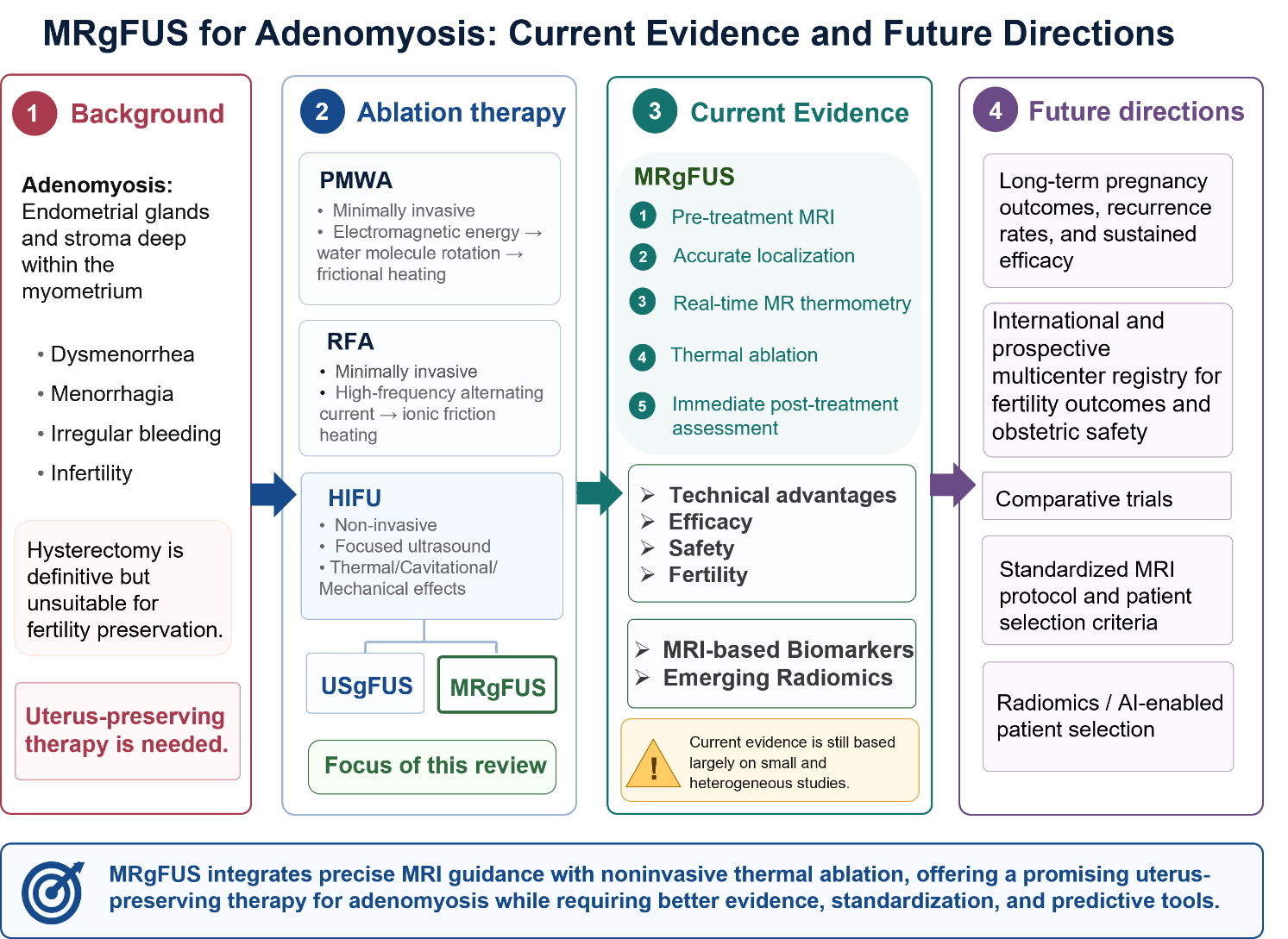

Supplement: Supplementary file 1 [file Data_Sheet_1.docx]
